# Photoelectrocatalytic Hydrogen Generation Enabled by CdS Passivated ZnCuInSe Quantum Dot-Sensitized TiO_2_ Decorated with Ag Nanoparticles

**DOI:** 10.3390/nano9030393

**Published:** 2019-03-08

**Authors:** Weili Li, Hongchao Geng, Lu Yao, Kesheng Cao, Pengtao Sheng, Qingyun Cai

**Affiliations:** 1 College of Chemistry and Environmental Engineering, Pingdingshan University, Pingdingshan 467000, China; genghc@126.com (H.G.); lucy94yao@126.com (L.Y.); 18737532760@126.com (K.C.); hnshengpengtao@126.com (P.S.); 2 State Key Laboratory of Chemo/Biosensing and Chemometrics, Hunan University, Changsha 410082, China; 3 College of Chemistry and Molecular Engineering, Zhengzhou University, Zhengzhou 450000, China

**Keywords:** ZCISe QDs, TiO_2_ nanowires, photocatalytic, hydrogen generation

## Abstract

Here we present the photoelectrocatalytic hydrogen generation properties of CdS passivated ZnCuInSe (ZCISe) quantum dots (QDs) supported by TiO_2_ nanowires decorated with Ag nanoparticles. In this configuration, Ag nanoparticles were sandwiched between the photo-electrons collector (TiO_2_) and photo-sensitizers (ZCISe), and acted as an electron relay speeding up the charge carrier transport. ZCISe and CdS enabled the optical absorption of the photoelectrode ranging from ultraviolet to near infrared region, which significantly enhanced the solar-to-chemical energy conversion efficiency. A photocurrent of 10.5 mA/cm^2^ and a hydrogen production rate of about 52.9 μmol/h were achieved under simulated sunlight (1.5 AG).

## 1. Introduction

Hydrogen generation by solar energy water splitting with a semiconductor photocatalyst is an interesting way to use renewable resources [1,2,3]. The solar-to-chemical energy conversion efficiency for a given photocatalyst is heavily dependent on light absorption and the rate between charge carriers separation (or migration to the surface) and electron-hole recombination [4,5,6,7], with an ideal semiconductor photocatalyst that presents high absorption and charge carriers separation. Hence, many semiconductor photocatalytic materials have been exploited for sustainable development. TiO_2_ is still the most widely used semiconductor due to its chemical stability, low cost, and good catalytic activity. For instance, Minero studied the relationship between light absorption and degradation rate in four TiO_2_ specimens, which was helpful for the degradation of formic acid at a low concentration [8]. TiO_2_ was also employed by Emeline to research the effects of light intensity and phenol concentration on the degradation [9]. Lasa researched plenty of work on photocatalysis and concluded that TiO_2_ photocatalysis also depended on the crystal structure of TiO_2_, cocatalyst, substrates, and sacrificial agents [10]. Using the sol-gel method, Rusinque and co-worker prepared mesoporous TiO_2_ for photocatalysis and hydrogen production reaching up to 210 cm^3^ STP (standard temperature and pressure) when using the 1.00 wt%-Pd on TiO_2_ [11]. However, TiO_2_ can only absorb the UV light due to wide bandgap, therefore limiting the overall efficiency [12,13]. In order to extend its light absorption into the visible light region, a narrow bandgap semiconductor combined with TiO_2_ has been used to form a heterostructure [14]. I-III-VI_2_ group QDs, such as CuInS_2_, CuInSe_2_, AgInS_2_, AgInSe_2_, etc., displayed narrow band gap, small self-absorption, large coefficient, and luminescence of the near-infrared wavelength region, and they have been successfully utilized in photocatalysis [15]. To adjust the light-harvesting ability and efficiency, various doped QDs were developed, such as Zn-Cu-In-Se, CuInSe_x_S_2−x_, and Cu-In-Ga-Se [16,17,18]. Zhong’s group doped CuInSe (CISe) QDs with the Zn ion, and successfully located the absorption spectra at a near-infrared region, demonstrating a PCE (photoelectric conversion efficiency) of 11.6% [18]. Subsequently, efficiency was further enhanced to 12% using the nitrogen-doped mesoporous carbon counter electrode in ZnCuInSe (ZCISe) QDs sensitized solar cells [19]. Inspired by such remarkable performance in photo-electricity conversion, we improved the photocatalytic H_2_ generation efficiency in a photo-electrochemical cell by placing the relatively less novel silver nanoparticles (Ag NPs) between the photo-electrons collector of one-dimensional TiO_2_ nanowires and the photo-sensitizers of CdS passivated ZCISe QDs. In such a configuration, Ag NPs can not only efficiently reserve the photo-generated hot electrons and reduce charge recombination, thereby enhancing photocatalytic activity [20], but also work with engineered optical and catalytic properties to activate and speed up steady-state photocatalytic reactions [21]. 

## 2. Experimental Section

### 2.1. Chemicals

Se powder (200 mesh, 99.99%), copper chloride (CuCl, 99.998%), Oleylamine (OAm, 80%–90%), 1-Dodecanethiol (DDT, 98%), and 1-octadecene (ODE, 90%) were purchased from Aladdin. Indium(III) chloride tetrahydrate (InCl_3_.4H_2_O, 99.99%) were received from Energy Chemical. Zinc chloride (ZnCl_2_, 99.9%) was obtained from Alfa Aesar (Zhengzhou, China). All chemicals were used as received without further processing. Deionized water with a resistance of 18.3 MΩ·cm was used in the experiment.

### 2.2. Preparation of ZCISe QDs

The ZCISe QDs were prepared by a hot-injection technique [18]. A mixture containing 3 mL DDT, 1 mL ODE, and 5 mL OAm was added to a 20 mL three-neck flask containing ZnCl_2_ (0.05 mmol), CuCl (0.03 mmol), and InCl_3_.4H_2_O (0.1 mmol) under a nitrogen atmosphere. The mixture was heated to 160 °C at a rate of 15 °C/min. Then, the Se (Se powder, 0.3 mmol) precursor solution with 0.1 mL OAm and 1 mL DDT, was injected into the flask. After remaining at 160 °C for a definite amount of time (10 min, 20 min, 30 min), the mixture was cooled in an ice bath. After 20 min, the nanocrystals were precipitated with excess ethanol and centrifuged at 6000 rpm for 5 min. Then, the supernatant was discarded, and the precipitate was redispersed with 3 mL dichloromethane. After centrifugation at 10,000 rpm for 10 min, the supernatant was collected and stored at 4 °C.

### 2.3. Decoration of Ag NPs on TiO_2_ Nanowires 

TiO_2_ nanowires were synthesized based on our previous method [22]. For the decoration of Ag NPs, TiO_2_ nanowires were immersed in 0.002 M AgNO_3_ aqueous solution and exposed to ultraviolet light at different times (5 min, 10 min, 15 min, 20 min, 25 min) [23]. Then, the Ag NPs loaded on the TiO_2_ nanowires were washed with ultrapure water three times.

### 2.4. Loading ZCISe QDs on TiO_2_/Ag

ZCISe QDs were decorated on the TiO_2_/Ag nanowires by dropping 20 μL ZCISe QD solution in CCl_3_ onto the nanowires, and then dried in an oven at 50 °C. This process can be repeated several times to get the optimal loading of ZCISe QDs. Finally, the electrode was annealed at 450 °C in an N_2_ flow for 30 min to improve the crystalline and stability.

### 2.5. Fabrication of TiO_2_/Ag/ZCISe/CdS Nanocomposite

CdS NPs were deposited on the TiO_2_/Ag/ZCISe composite with a successive ionic layer adsorption and reaction (SILAR) method to decrease the surface defects of the ZCISe QDs. In brief, 0.05 M cadmium nitrate in ethanol was used as cation source, and 0.05 M sodium sulfide in methanol/water (1/1) was used as anion source. The TiO_2_/Ag/ZCISe nanocomposite was immersed in Cd precursor for one minute followed by flushing with ethanol, and then immersed in sodium sulfide precursor for another minute followed by rinsing with methanol. This counted as one SILAR cycle. For the present work, this process was repeated for 5 cycles.

### 2.6. Characterization

UV-vis absorption measurements were obtained on a Shimadzu-NIR-2550 spectrophotometer (Guangzhou, China). Photoluminescence (PL) spectra were recorded on a Hitachi F-7000 fluorescence spectrophotometer (Hitachi High-Tech, Tokyo, Japan). The structure of the ZCISe QDs was identified via an X-ray diffractometer (XRD) on a D8 advance (Bruker, AXS, Madison, WI, USA) with Cu–Ka radiation. The morphology of the sample was characterized by scanning electron microscopy (SU8010, Hitachi, Hong Kong, China) and transmission electron microscopy (Tecnai G^2^ F20 S-Twin, FEI, Hillsboro, OR, USA). Elemental analysis was carried out on an energy disperse X-ray spectrometer (EDX) fitted with TEM. The performances were measured with a CHI electrochemical workstation (CHI660E, Shanghai Chenhua Instrument Co. Ltd., Shanghai, China). The light source was a 300 W Xe lamp equipped with a band pass filter at 500 nm with a bandwidth (full width at half maximum) of 15 nm to remove IR and UV radiations, and a cutoff filter for eliminating radiation below 800 nm, respectively.

### 2.7. Photoelectrochemical Performance and Hydrogen Generation Test

The photoelectrochemical test was carried out in a standard three-electrode system composed of an Ag/AgCl reference, a Pt sheet counter electrode, and samples of the working electrode in an electrolyte containing 0.35 M Na_2_SO_3_ and 0.24 M Na_2_S, which was purged with N_2_ for 30 min prior to measurements. The incident light from a 300 W Xe lamp was filtered to match the AM 1.5 G spectrum. The hydrogen production test was essentially the same as the material’s performance test, except the electrolyte was replaced with pure water. The amount of hydrogen gas generated during 6 h of irradiation was collected in a home-made device [24].

## 3. Results and Discussion

### 3.1. Characterization of ZCISe QDs

**Scheme 1:**
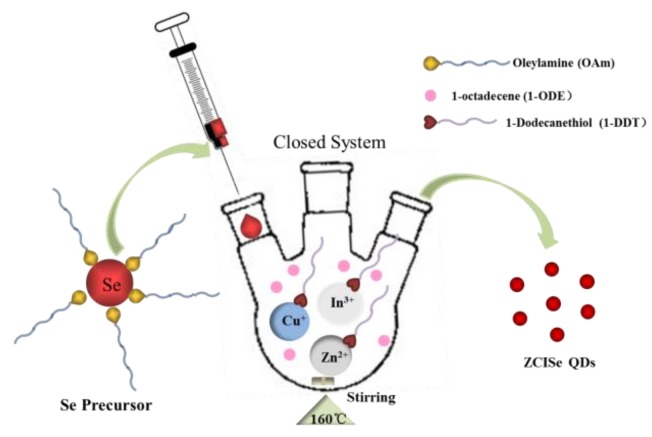
The synthesis schematic diagram of ZCISe QDs.

An injection route was used to synthesize the Zn-Cu-In-Se QDs (Scheme 1). Firstly, zinc, copper, and indium chlorides were solubilized in ODE using OAm, which prevented slow nucleation and rapid aggregation of the resulting materials, and DDT reduced the colloidal stability of the QDs particularly at high temperatures as ligands. Additionally, the presence of DDT was critical to the synthesis. According to the theory of Hard–Soft–Acids–Base, the reactive Cu^+^ ion, a soft acid, is preferentially complexed by dodecanethiol, a soft base [25,26]. After degassing, the solution was heated under nitrogen. Then we observed the change of solution from turbid to clear and finally to light yellow, which indicated the metal chloride was dissolved. Meanwhile, Se powder was solubilized in OAm and DDT ligands. After injecting the Se precursor into the flask, the color of the solution changed to black red immediately. As the reaction time increased, it first turned bright red and then turned black, which indicated nanocrystal nucleation. 

**Figure 1:**
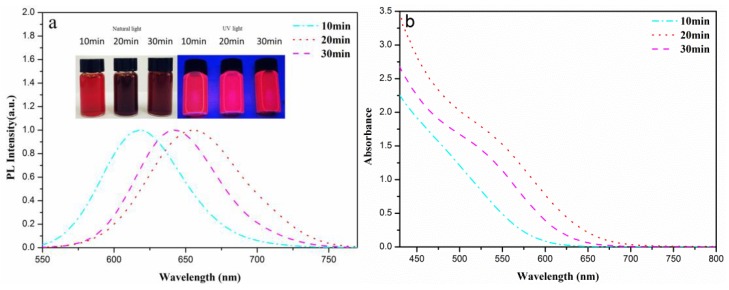
(**a**) Photoluminescence (PL) emission and (**b**) UV-vis absorption spectra of ZCISe QDs. The inset of panel (a) shows the digital photographs of the corresponding QDs under natural light and UV light illumination (365 nm).

Figure 1 shows the PL emission and UV-Vis absorption spectra of ZCISe QDs with different reaction times at 160 °C, with the corresponding PL emission photographs shown in the inset of Figure 1a. The PL emission underwent a red shift with the increase of reaction time to 20 min, and the largest absorption edge at about 670 nm. Meanwhile, the QDs with the reaction time of 20 min had the best absorption. Here we used ZCISe QDs with 20 min reaction time as the model QDs in the following experiments.

**Figure 2:**
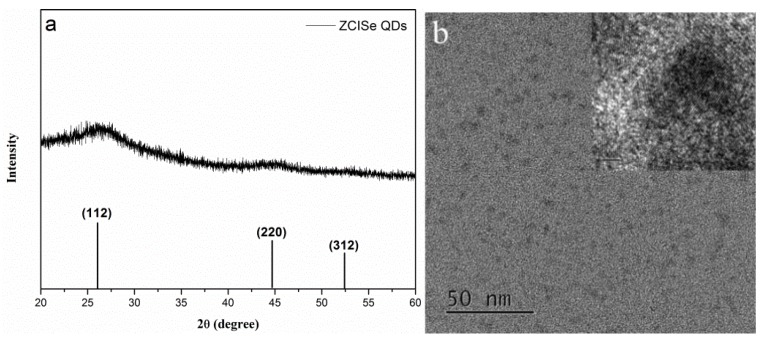
(**a**) XRD, (**b**) TEM and HRTEM (inset of (**b**)) images of the ZCISe QDs.

The XRD pattern of ZCISe QDs prepared with 20 min reaction time is shown in Figure 2a. The diffraction peaks at 2θ of 26.77°, 44.43°, and 54.64° are assigned to the (112), (220), and (312) crystal planes of the chalcopyrite (tetragonal) ZCISe QDs (JCPDS 00-051-1221), respectively. The wider peaks represent the small size of ZCISe QDs. Meanwhile, the transmission electron microscopy (TEM) images (Figure 2b) and high-resolution electron microscopy (HRTEM) (inset of Figure 2b), show that the ZCISe QDs are identical with an average particle size of 4.1 ± 0.3 nm. The homogeneous size distribution in the TEM observation is consistent with the distinct excitonic peak observed in the absorption spectra (Figure 1b) and calculated size from XRD.

### 3.2. Characterization of TiO_2_/Ag/ZCISe/CdS Nanocomposite

**Figure 3:**
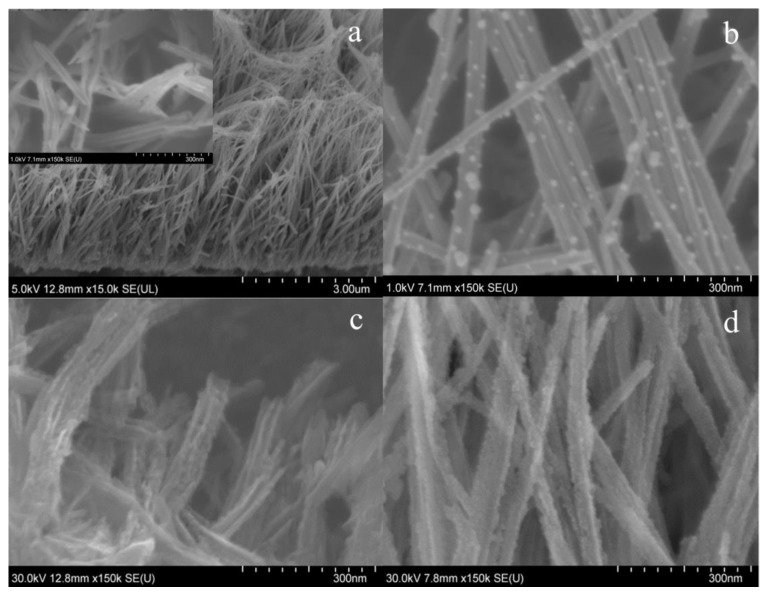
The SEM images of TiO_2_ nanowire arrays (**a**), TiO_2_/Ag (**b**), TiO_2_/Ag/ZCISe (**c**) and TiO_2_/Ag/ZCISe/CdS (**d**).

The TiO_2_ nanowires are a smooth porous network with the top loosely arranged with an average diameter and length corresponding to 40 nm and 3 μm, respectively, as shown in Figure 3a. High magnification of the TiO_2_ nanowires is shown in the inset of Figure 3a. After subsequently assembling with Ag NPs (Figure 3b) and ZCISe QDs (Figure 3c), lots of tiny particles anchor onto the TiO_2_ nanowires which consequently turn rough. Figure 3d represents the SEM image of TiO_2_/Ag/ZCISe/CdS. One can see that TiO_2_/Ag/ZCISe are almost completely coated by a thin CdS layer without the naked TiO_2_ surface, which can greatly decrease the surface defects that are related to the charge carriers’ recombination. 

**Figure 4:**
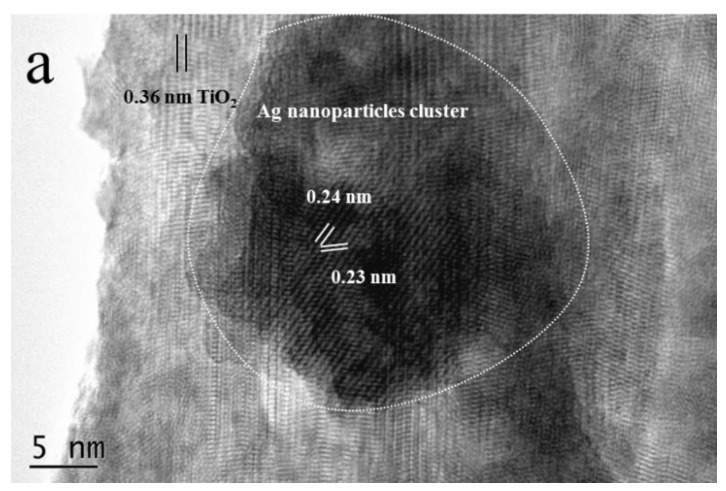

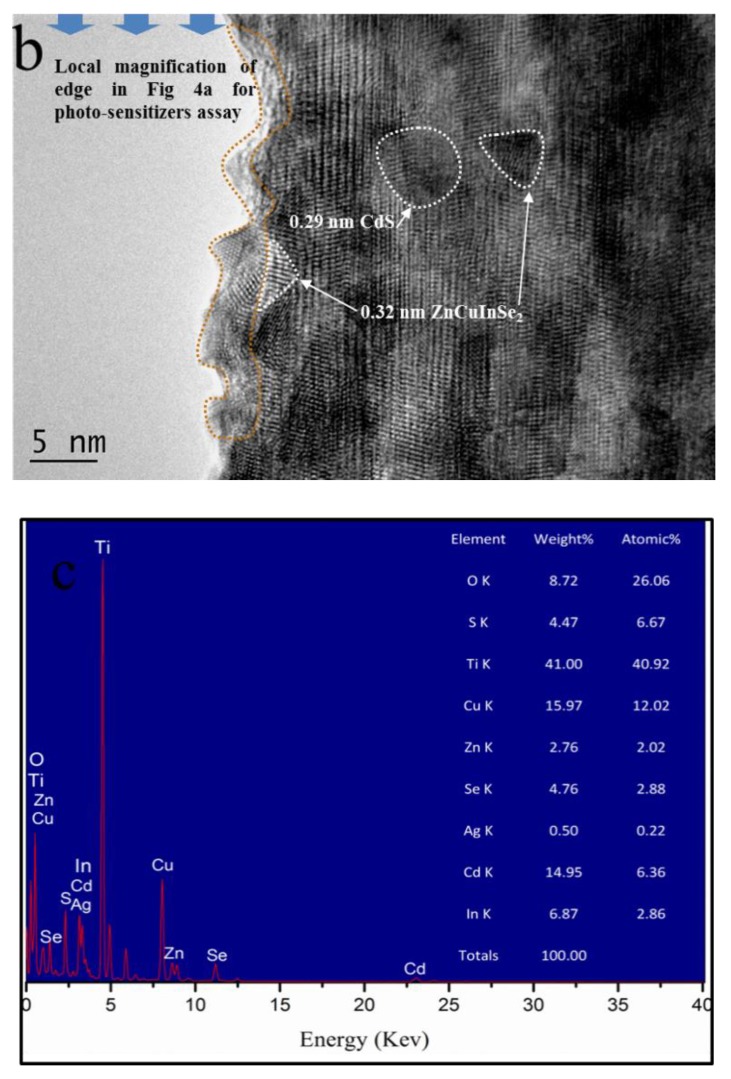
(**a**) HRTEM, (**b**) Local magnification and (**c**) EDS images of TiO_2_/Ag/ZCISe/CdS.

The HRTEM images in Figure 4a,b show that the Ag nanoparticles, ZCISe QDs, and CdS nanoparticles are tightly grown on the surface of the TiO_2_ nanowires. The HRTEM image with clear lattice spaces of TiO_2_, Ag, ZCISe, and CdS displays high crystallinity of the as-synthesized TiO_2_/Ag/ZCISe/CdS composite in Figure 4b. The EDS spectrum of Figure 4c shows the characteristic peaks of O, S, Ti, Cu, Zn, Se, Ag, Cd, and In elements.

### 3.3. Optical Absorption

**Figure 5:**
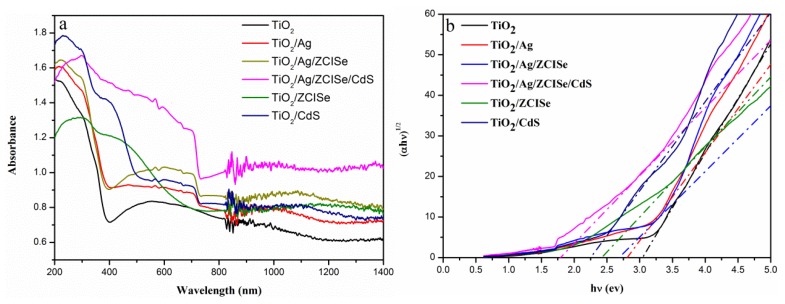
(**a**) UV-Vis spectra of TiO_2_, TiO_2_/Ag, TiO_2_/ZCISe, TiO_2_/CdS, TiO_2_/Ag/ZCISe, and TiO_2_/Ag/ZCISe/CdS composite structure, (**b**) the Tauc plots of different samples.

Figure 5a shows the UV-vis spectra of the TiO_2_ nanowires, TiO_2_/Ag, TiO_2_/Ag/ZCISe, and TiO_2_/Ag/ZCISe/CdS. After the sensitization of ZCISe QDs, the absorption spectrum of the TiO_2_ nanowires was significantly enhanced. The TiO_2_/Ag/ZCISe/CdS electrode exhibited red-shift absorption at a wavelength range between 680 and 1000 nm. The surface passivation can enhance the absorption spectra towards long wavelength region compared with those of pure TiO_2_ and TiO_2_/Ag/ZCISe. This red-shift may indicate that the QDs charge carriers wave functions tunnel into the surrounding of the CdS shell. UV-Vis spectra were also measured to reveal the band gaps of the as-prepared samples, using Tauc relation [27,28],

(αhν)^1/2^ = A(hν − Eg) (1)

where α is the light absorption coefficient, A is a constant, h is the Planck’s constant, ν is light frequency, and Eg is the band gap energy. The optical band gaps of TiO_2_/Ag/ZCISe/CdS, TiO_2_/Ag/ZCISe, and TiO_2_/Ag correspond to 1.75 eV, 2.7 eV, and 2.8 eV in Figure 5b.

### 3.4. Electrochemical Characterization

**Figure 6:**
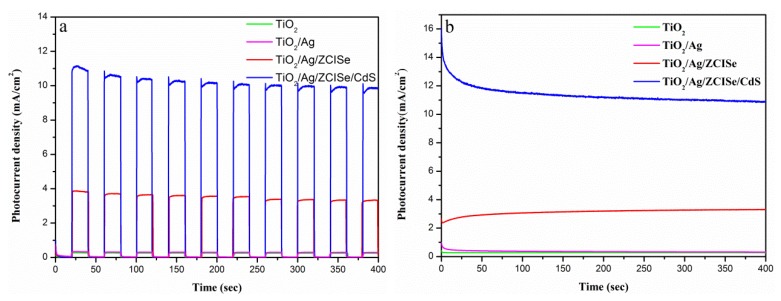
The photocurrent density of electrodes under intermittent (**a**) and successive (**b**) simulated sunlight irradiation.

A three-electrode system was used to determine the photocurrent density of different electrodes. It is well known that the optimized photoreduction deposition time of Ag nanoparticles benefit to enhance the photocurrent density. The photocurrent densities of TiO_2_/Ag monotonically increased with the increasing of Ag loading content corresponding to the reaction time, whereas the photocurrent density sharply decreased as the reaction time reached 20 min. Fifteen minutes was chosen for the single-step photoreduction deposition time to improve the photoelectrochemical properties as shown in Figure S1A. Notably, by characterizing the SEM images of TiO_2_/Ag with different deposition times (Figure S2, Detailed Analysis), the obtained results were consistent with the change trend of the photocurrent density. In addition, the amount of ZCISe QDs and CdS for the photocurrent density also played a key role in the proposed photoelectrode. The photocurrent density of TiO_2_/Ag/ZCISe and TiO_2_/Ag/ZCISe/CdS increased and then decreased with the increase in the quantity of ZCISe and the number of layers covered by CdS (Figure S1B,C). Due to the excessive load which interfered with the absorption of light by the photoelectrodes, the optimal quantity and the number of cycles were 1 mL and 5 times, respectively. Figure 6a,b shows the I-t curves of TiO_2_, TiO_2_/Ag, TiO_2_/Ag/ZCISe, and TiO_2_/Ag/ZCISe/CdS ptotoelectrodes. It can be seen that the photocurrent density of TiO_2_/Ag displayed a small increase compared with the pristine TiO_2_ nanowires. After loading the ZCISe QDs, the photocurrent density increased about 10 times, reaching 3.66 mA/cm^2^. Further loading the CdS shell, the photocurrent density increased about 2.8 times more than TiO_2_/Ag/ZCISe, reaching 10.5 mA/cm^2^. In order to determine the function of each part in the proposed photoelectrode, the photocurrent densities of different modified photoelectrodes were measured under chopped illumination of AM 1.5 G light with a cutoff filter and a band pass filter for obtaining the different wavelength as shown in Table S1. Under the visible light (500 nm ± 15 nm), ZCISe, Ag/ZCISe, and CdS modified TiO_2_ exhibited the largest photocurrent densities. TiO_2_/ZCISe and TiO_2_/Ag/ZCISe also had a non-negligible response in the IR region, indicating the role of ZCISe QDs in absorbing IR. However, due to the lower intensity of IR light, the obtained photocurrent density were less than the above results. After connecting the parts together, the proposed photoelectrode showed an excellent photoelectrochemical performance in accordance with the UV-vis spectra (Figure 5).

### 3.5. Photocatalytic Performance of Hydrogen Generation

**Figure 7:**
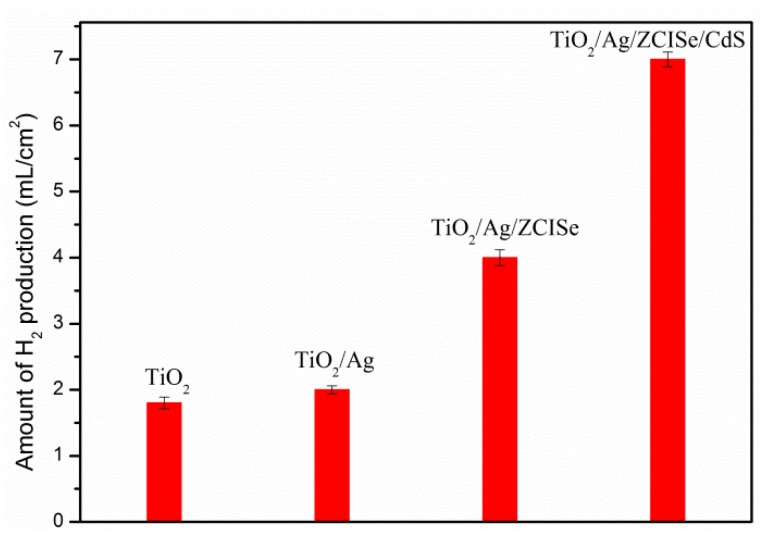
Hydrogen production of different materials.

The photocatalytic activities of the as-prepared TiO_2_, TiO_2_/Ag, TiO_2_/Ag/ZCISe, and TiO_2_/Ag/ZCISe/CdS photoelectrodes were evaluated by hydrogen production under high-power xenon lamp irradiation. As shown in Figure 7, after 6 h irradiation by the xenon lamps, the H_2_ yields by the different photoelectrodes were about 1.8, 2, 4, and 7 mL, respectively. Notably, the related photocatalytic rate constant of hydrogen evolution by TiO_2_/Ag/ZCISe/CdS reached 52.9 μmol/h, which was approximately 3.88 times of that by the photoelectrode of pure TiO_2_ nanowires. This is due to the sensitization of ZCISe QDs which broadens the light absorption range of the TiO_2_/Ag/ZCISe/CdS electrode, increases the separation efficiency of electrons-holes, and generates more electrons. The CdS passivates the surface of the QDs and inhibits the recombination of electron holes. In addition, the decoration of Ag NPs promoted the transmission of electrons and enhanced the photocatalytic activity.

### 3.6. Mechanism of Charge Transfer in TiO_2_/Ag/ZCISe/CdS

**Figure 8:**
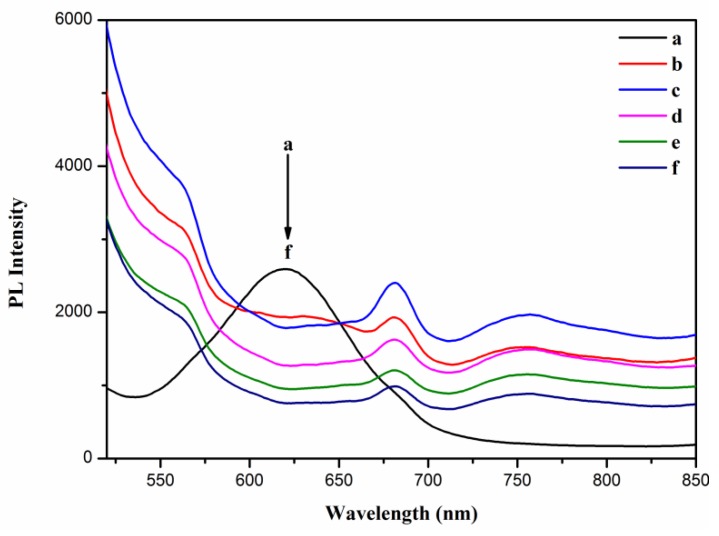
PL spectrum of (**a**) FTO/ZCISe, (**b**) TiO_2_/ZCISe, (**c**) FTO/ZCISe/CdS, (**d**) TiO_2_/ZCISe/CdS, (**e**) TiO_2_/Ag/ ZCISe, (**f**) TiO_2_/Ag/ZCISe/ CdS.

Based on the photoelectric characterization of composite electrode and hydrogen production performance, the mechanism of charge transfer in TiO_2_/Ag/ZCISe/CdS was discussed using PL emission spectra as shown in Figure 8. When ZCISe QDs were loaded onto TiO_2_ nanowires, a distinct quenching of the emission was observed when compared with the composite structure of FTO/ZCISe(FTO, The SnO_2_ transparent conductive glass of doped F.), which demonstrated deactivation of the excited ZCISe via electron transfer to the TiO_2_. In the same way, electrons can be transferred from CdS NPs to ZCISe QDs and then to TiO_2_ nanowires by comparing FTO/ZCISe/CdS and TiO_2_/ZCISe/CdS. Moreover, the emission further significantly quenched when Ag NPs were decorated between TiO_2_ nanowires and ZCISe to form the TiO_2_/Ag/ZCISe sandwich, suggesting that pronounced charge separation occurred within the TiO_2_/Ag/ZCISe. The PL quenching of the composite agrees with an initial expectation of high efficient charge carriers transport pathway.

**Scheme 2:**
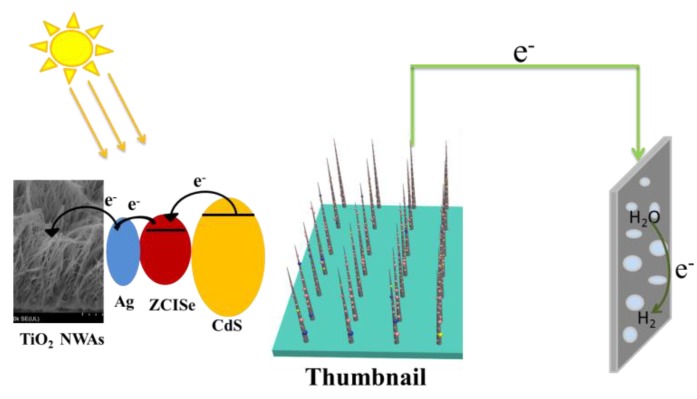
Schematic diagram of TiO_2_/Ag/ZCISe/CdS photoelectrode and its mechanism of photocatalytic hydrogen production.

On the basis of the results and discussion above, the schematic diagram and photocatalytic mechanism for the TiO_2_/Ag/ZCIS/CdS photoelectrode is proposed and schematically exhibited in Scheme 2. Under solar irradiation, CdS, ZCISe QDs, and TiO_2_ absorbed the light and excited. Electron-hole pairs are generated on CdS/ZCISe QDs, and the electrons are quickly transferred into the conduction band of TiO_2_ nanowires through Ag NPs. Then, electrons interact with H_2_O to produce hydrogen.

## 4. Conclusions 

In summary, oil-soluble ZCISe QDs were prepared by a simple hot-injection method, and a promising strategy with enhanced photocatalytic efficiency of CdS NPs, ZCISe QDs, and Ag NPs-based sandwich electrode configuration is present. The excellent architecture of TiO_2_/Ag/ZCISe/CdS showed good light-harvesting behavior and notably promoted photocatalytic hydrogen production rate of about 52.9 μmol/h. It is believed that the positive effect between Ag, ZCISe, and CdS as the components of co-catalyst on the photocatalytic hydrogen production activity, efficiently suppress charges recombination, improve interfacial charge transfer, and eventually provide good photocatalytic reaction centers. 

## Supplementary Materials

The following are available online at https://www.mdpi.com/2079-4991/9/3/393/s1, Figure S1: The dependence of photocurrent on the loaded Ag nanoparticals, ZCISe and CdS amount which is represented by the ultraviolet light exposure time (**A**), the amount of dropwise (**B**) and the number of soak (**C**) of the corresponding electrode, Figure S2: SEM images of TiO_2_/Ag with photoreduction deposition time of 5 min (A,A_1_), 10 min (B,B_1_), 15min (C,C_1_), 20 min (D,D_1_) and 25 min (E,E_1_) with different magnification, Table S1: Photocurrent density of different modified photoelectrode under three condition.
