# Supplementary material for: Photoelectrocatalytic Hydrogen Generation Enabled by CdS Passivated ZnCuInSe Quantum Dot-Sensitized TiO2 Decorated with Ag Nanoparticles
Source: Nanomaterials (Basel). 2019 Mar 8;9(3):393. doi: 10.3390/nano9030393 (PMC6474140; doi:10.3390/nano9030393)
Supplement: Supplementary file 1 [file nanomaterials-09-00393-s001.pdf]

Supporting Information

Photoelectrocatalytic Hydrogen Generation Enabled by CdS passivated ZnCuInSe Quantum Dot-Sensitized TiO_2 Decorated with Ag Nanoparticles

Weili Li[a]*, Hongchao Geng[a, b], Lu Yao[a, c], Kesheng Cao[a], Pengtao Sheng[a], Qingyun Cai[b]*

[a] College of Chemistry and Environmental Engineering, Pingdingshan University, Pingdingshan, 467000 China

[b] State Key Laboratory of Chemo/Biosensing and Chemometrics, Hunan University, Changsha, 410082 China

[c] College of Chemistry and Molecular Engineering, Zhengzhou University, Zhengzhou 450000, China

Corresponding Author E-mail: liweiliziji11@126.com, caigroup@hnu.edu.cn

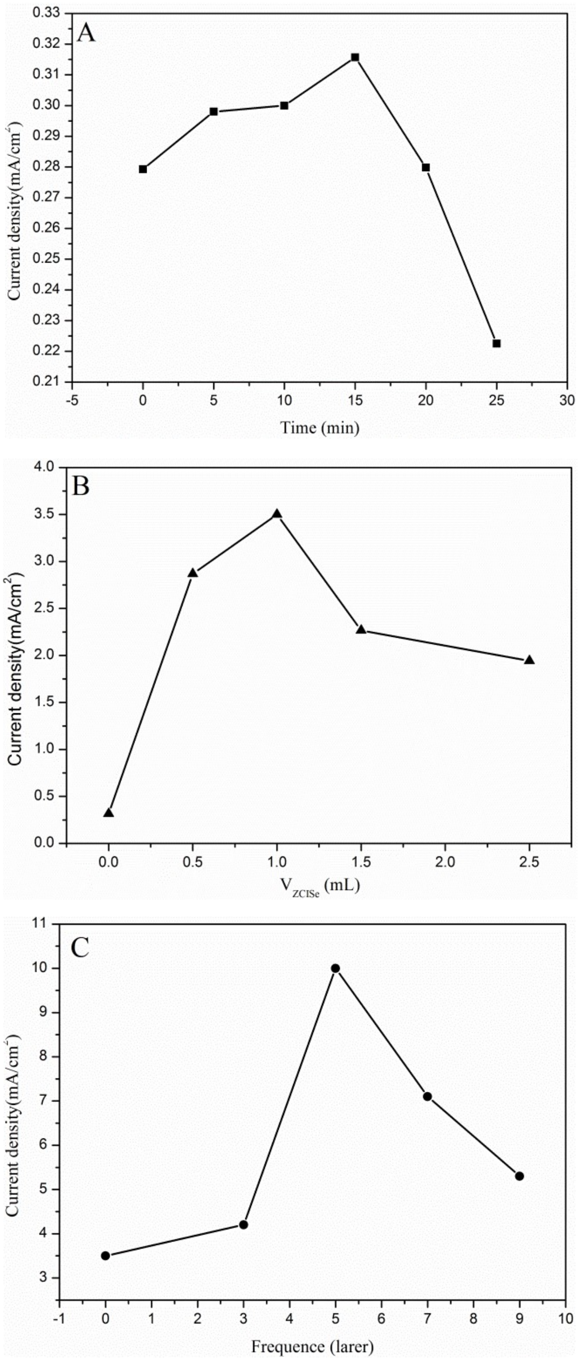

Figure S1. The dependence of photocurrent on the loaded Ag nanoparticals, ZCISe and CdS amount which is represented by the ultraviolet light exposure time (A), the amount of dropwise (B) and the number of soak (C) of the corresponding electrode.

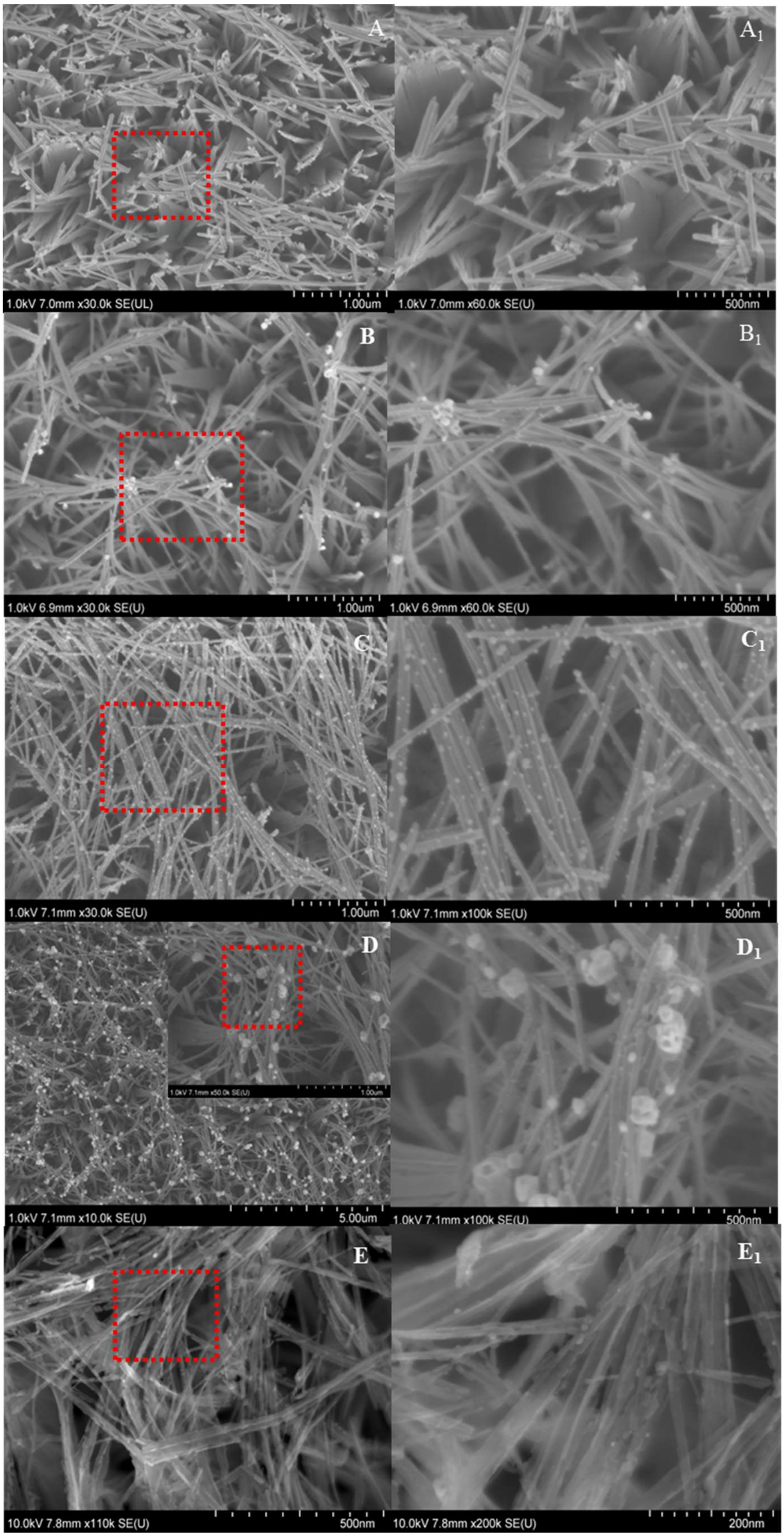

Figure S2. SEM images of TiO_2/Ag with photodeposition time of 5 min (A,A_1), 10 min (B,B_1), 15min (C,C_1), 20 min (D,D_1) and 25 min (E,E_1) with different magnification.

In order to investigate the detailed structural and surface information on the TiO_2 nanowires, SEM of TiO_2/Ag nanowires under different irradiation time using UV light was done. From Figure S2, TiO_2/Ag nanowires display very rough surface morphologies compared to pure TiO_2 nanowires, and the amount of Ag nanoparticles increase gradually as the time of irradiation progresses (Figure S2A, Figure S2B, and Figure S2C). Meanwhile, the corresponding photocurrent intensity also increased gradually. Notably, after 15 min of irradiation, a layer of tiny Ag nanoparticles is uniformly deposited on the surface of TiO_2 nanowires. However, with increasing the reaction time, small weak agglomerates are formed as shown in Figure S2D and Figure S2E. When the time reaches 25 min, the primary Ag nanoparticles in the state of agglomeration are clearly observed, which will affect the photoelectric property. From the above, this result is consistent with the trend of photocurrent intensity.

Table S1. Photocurrent density of different modified photoelectrode under three condition

Irradiation range	Photocurrent density of different modified photoelectrode (mA/cm^2)					
	TiO_2	TiO_2/Ag	TiO_2/Ag/ZCISe	TiO_2/ZCISe	TiO_2/CdS	TiO_2/Ag/ZCISe/CdS
λ =500 ±15 nm	0.05	0.10	2.64	2.1	2.9	5.7
$\lambda \geq$ 800 nm	/	/	0.27	0.23	0.1	0.4
300 ≦ λ ≦ 2500 nm	0.28	0.32	3.66	3.2	6.0	10.5
